# Humans Can Visually Judge Grasp Quality and Refine Their Judgments Through Visual and Haptic Feedback

**DOI:** 10.3389/fnins.2020.591898

**Published:** 2021-01-12

**Authors:** Guido Maiello, Marcel Schepko, Lina K. Klein, Vivian C. Paulun, Roland W. Fleming

**Affiliations:** ^1^Department of Experimental Psychology, Justus Liebig University Giessen, Giessen, Germany; ^2^Center for Mind, Brain and Behavior, Justus Liebig University Giessen, Giessen, Germany

**Keywords:** grasping, visual grasp selection, precision grip, shape, material, motor imagery, action observation

## Abstract

How humans visually select where to grasp objects is determined by the physical object properties (e.g., size, shape, weight), the degrees of freedom of the arm and hand, as well as the task to be performed. We recently demonstrated that human grasps are near-optimal with respect to a weighted combination of different cost functions that make grasps uncomfortable, unstable, or impossible, e.g., due to unnatural grasp apertures or large torques. Here, we ask whether humans can consciously access these rules. We test if humans can explicitly judge grasp quality derived from rules regarding grasp size, orientation, torque, and visibility. More specifically, we test if grasp quality can be inferred (i) by using visual cues and motor imagery alone, (ii) from watching grasps executed by others, and (iii) through performing grasps, i.e., receiving visual, proprioceptive and haptic feedback. Stimuli were novel objects made of 10 cubes of brass and wood (side length 2.5 cm) in various configurations. On each object, one near-optimal and one sub-optimal grasp were selected based on one cost function (e.g., torque), while the other constraints (grasp size, orientation, and visibility) were kept approximately constant or counterbalanced. Participants were visually cued to the location of the selected grasps on each object and verbally reported which of the two grasps was best. Across three experiments, participants were required to either (i) passively view the static objects and imagine executing the two competing grasps, (ii) passively view videos of other participants grasping the objects, or (iii) actively grasp the objects themselves. Our results show that, for a majority of tested objects, participants could already judge grasp optimality from simply viewing the objects and imagining to grasp them, but were significantly better in the video and grasping session. These findings suggest that humans can determine grasp quality even without performing the grasp—perhaps through motor imagery—and can further refine their understanding of how to correctly grasp an object through sensorimotor feedback but also by passively viewing others grasp objects.

## Introduction

When we try to grasp objects within our field of view, we rarely fail. We almost never miss the object or have it slip out of our hands. Thus, humans can very effectively use their sense of sight to select where and how to grasp objects. Yet for any given object, there are numerous ways to place our digits on the surface. Consider a simple sphere of 10 cm diameter and ∼300 cm^2^ surface area. If we coarsely sample the surface in regions of 3 cm^2^ (a generous estimate of the surface of a fingertip) there are approximately 100 surface locations on which to place our digits. Even when considering simple two-digit precision grips, which employ only the thumb and forefinger, there are ∼10,000 possible digit configurations that could be attempted. How do humans visually select which of these configurations is possible and will lead to a stable grasp?

To answer this question, in recent work (Klein et al., 2020) we asked participants to grasp 3D polycube objects made of different materials (wood and brass) using a precision grip. Even with these objects—geometrically more complex than a simple sphere—participants consistently selected only a handful of grasp configurations, with different participants selecting very similar grasps. This suggests that a common set of rules constrains how people visually select where to grasp objects. We formalized this observation, following (Kleinholdermann et al., 2013), by constructing a computational model that takes as input the physical stimuli, and outputs optimal grasp locations on the surface of the objects. Specifically, we constructed a set of optimality functions related to the size, shape, and degrees of freedom of the human hand, as well as to how easily an object can be manipulated after having been grasped. Model predictions closely agreed with human data, demonstrating that actors choose near-optimal grasp locations following this set of rules.

The strongest constraint for two-digit grasps, included in this computational framework, requires surface normals at contact locations to be approximately aligned (a concept known as force closure; Nguyen, 1988). Fingertip configurations that do not fulfill this constraint, e.g., with thumb and forefinger pushing on the same side of an object, cannot lift and manipulate the object. Indeed successful human grasps never fail to meet the force closure constraint (Kleinholdermann et al., 2013; Klein et al., 2020). The other constraints we implemented as optimality functions relate to:

*Natural grasp axis*: humans exhibit a preferred hand orientation for precision grip grasping, known as the natural grasp axis (Roby-Brami et al., 2000; Lederman and Wing, 2003; Schot et al., 2010; Voudouris et al., 2010), which falls within the midrange of possible hand and arm joint angles. Grasps rotated away from the natural grasp axis may result in uncomfortable (or impossible) hand/arm configurations that require extreme joint angles. Since these extreme joint angles should be avoided (Rosenbaum et al., 2001), optimal grasps should exhibit minimum misalignment with the natural grasp axis.

*Grasp aperture*: When free to employ any multi-digit grasp, participants select precision grip grasps only when the required distance between finger and thumb at contact (the “grasp aperture”) is smaller than 2.5 cm (Cesari and Newell, 1999). As grasp size increases, humans progressively increase the number of digits employed in a grasp. Therefore, optimal two-digit precision grips should exhibit grasp apertures below 2.5 cm.

*Minimum torque*: grasping an object far from its center of mass results in high torques, which may cause the object to rotate when manipulated (Goodale et al., 1994; Lederman and Wing, 2003; Eastough and Edwards, 2006; Lukos et al., 2007; Paulun et al., 2016). Large gripping forces would be required to counteract high torques and prevent the object from rotating. Thus, optimal grasps should have minimum torque.

*Object visibility*: when grasping an object, the hand might occlude part of an object from view. This could be detrimental for subsequent object manipulation, and indeed humans exhibit spatial biases in their grasping behavior which are consistent with avoiding object occlusions (Paulun et al., 2014; Maiello et al., 2019). Therefore, optimal grasps should minimize the portion of an object occluded from view.

Whereas the force closure constraint is necessary and immutable, the relative importance given to the other four constraints varies with object properties (e.g., mass) and across participants.

Given the computational costs, it seems relatively unlikely that the brain fully computes these optimality functions for every possible grasp. Nevertheless, our previous findings suggest that humans can employ visual information to estimate these constraints and guide grasp selection. As a further test of our framework for understanding human grasp selection, here we ask whether human participants can explicitly report relative grasp optimality (i.e., which of two candidate grasps would be closer to optimal). We further ask whether observers can judge grasp optimality using vision alone, or whether executing a grasp is necessary to do so.

If participants were indeed better at judging grasp optimality when executing grasps, this might suggest that tactile (Johansson and Westling, 1984) and proprioceptive feedback from our arm and hand (Rosenbaum et al., 2001; Lukos et al., 2013) plays a role in evaluating grasp quality. Humans may employ these sources of feedback to learn that certain hand configurations are uncomfortable, or that one grasp requires more force than another to pick up the same object.

Additionally, participants might also be able to visually assess the characteristics of their own movements, such as the speed and trajectory of the limb. Previous work has in fact demonstrated that humans can access visual information of grasping kinematics. For example, human participants can estimate the size (Campanella et al., 2011; Ansuini et al., 2016) and weight (Podda et al., 2017) of unseen objects by observing the reach to grasp movements performed by others. These sources of visual information are known to play a strong role in grasp planning and execution, as removing them changes the kinematics of grasping movements (Connolly and Goodale, 1999), and even simply observing others execute grasping tasks can improve one’s own grasping performance (Buckingham et al., 2014). We therefore, ask how much these sources of visual information might contribute to participant judgements of grasp optimality. Specifically, we test whether grasp quality can be inferred from watching grasps executed by others. If this were the case, then perhaps vision and proprioception may be redundant sources of information about grasp quality, which could aid humans in linking vision and motor control in action planning.

To test whether humans can explicitly judge grasp quality, in Experiment 1 we asked participants to report which of two candidate grasps on an object is best, first using vision alone (vision session), and then also by attempting both grasps on the object, one after the other (grasping session). To test whether visual information about the grasping movements plays a role in judging grasp quality, in Experiment 2a we asked a new set of participants to repeat a subset of key conditions from Experiment 1, while we video-recorded their grasping movements. Finally, in Experiment 2b we showed these recorded movements to yet another set of participants (video session), and asked them to judge grasp quality from the videos of grasps executed by participants from Experiment 2a.

## Materials and Methods

### Participants

We recruited 21 naïve and right-handed participants [16 female, 5 male; mean (range) age: 24 (19–32) years] for Experiment 1, 25 naïve and right-handed participants [17 female, 8 male; mean (range) age: 23 (20–26)] for Experiment 2a, and 25 naïve and right-handed participants [18 female, 7 male; mean (range) age: 24 (19–36)] for Experiment 2b. Participants were staff and students from Justus Liebig University Giessen, Germany. In return for their participation, volunteers were paid 8 EURO per hour. Participants reported healthy upper extremities and normal or corrected to normal vision. All provided written informed consent. All procedures were approved by the local ethics committee of Justus Liebig University Giessen (Lokale Ethik−Kommission des Fachbereichs 06, LEK−FB06; application number: 2018-0003) and adhered to the tenets of the declaration of Helsinki.

### Apparatus

**FIGURE 1:**
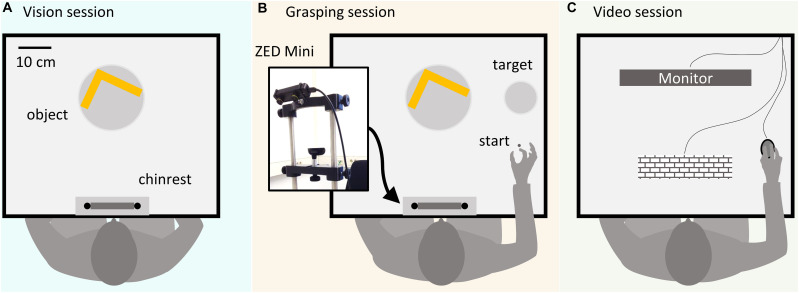
Schematic representation of the experimental setup. **(A)** In the vision sessions participants passively viewed objects and evaluated the relative optimality of preselected grasps without executing the grasps. **(B)** In the grasping sessions, participants executed grasps prior to judging the grasp quality. A ZED Mini stereo camera positioned above the participant’s forehead recorded the grasping movements in Experiments 2a and 2b. **(C)** In the video session, participants from Experiment 2b viewed recordings of grasps executed by participants from Experiment 2a on a computer monitor.

All Experiments (1, 2a, 2b) were programmed in Matlab version 2018a. Participants were seated at a table with a mounted chin rest in a brightly lit room. Figure 1 shows a schematic of the setup. In all experiments, during the vision (Figure 1A) and grasping sessions (Figure 1B), subjects positioned their heads in the chinrest before each trial. Stimulus objects were positioned 34 cm in front of the participant. At this predefined position, a turntable allowed the experimenter to precisely set object orientation. In the grasping sessions, participants were instructed to grasp the objects and move them to a target location shifted 23 cm to the right side from the initial object location along the horizontal axis, at a distance of 40 cm relative to the participant. The starting position for the right thumb and index finger was 24 cm to the right and 22 cm in front of the participant. In grasping sessions, objects were grasped with a precision grip at two predetermined locations. A ZED Mini stereo camera (Stereolabs) was attached to the front of the forehead rest to record (720p, 30 fps) grasping movements in Experiments 2a and 2b. To record videos, a simple recording program was written in C++, using the ZED SDK, and called from within the Matlab environment. The camera orientation was adjustable along the frontal axis and fixed at a downwards tilt angle of 25° to capture the whole movement sequence. During the experiment, participants did not see the camera due to its position right in front of their forehead (Figure 1B). In Experiment 2b (Figure 1C), videos were presented on an Asus VG248QE monitor (24″, resolution = 1,920×1,080 pixel) at 60 Hz, positioned at a distance of 40 cm from the observers.

### Experiment 1

#### Stimuli

**FIGURE 2:**
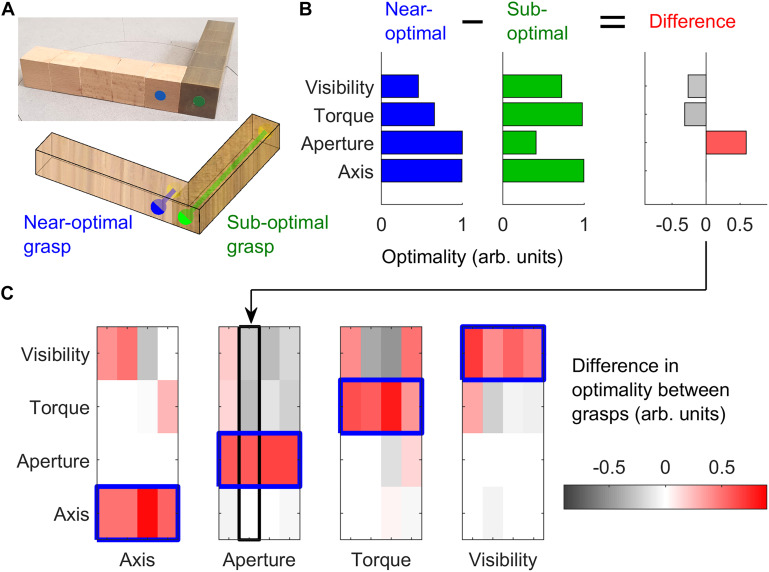
Stimulus selection. **(A)** One example object in which we selected one optimal (blue) and one sub-optimal (green) grasp with respect to grasp aperture. The right side of the object is made of brass, the left side of beech wood. Blue and green dots represent thumb contact locations; the index finger is to be placed on the opposing surface. The blue grasp requires a small (2.5 cm) grip aperture, and is thus optimal with respect to grasp aperture. The green grasp requires a large grip aperture (12.5 cm) and is thus sub-optimal. **(B)** For the two selected grasps in panel **(A)**, we plot the optimality of the grasps (in normalized, arbitrary units) for each of the 4 optimality criteria, and the difference in optimality between grasps. **(C)** The difference in grasp optimality is shown for all pairs of grasps selected on all 16 objects, 4 per optimality criteria. Red indicates the selected near-optimal grasp is better than the selected sub-optimal grasp. Each column corresponds to one of the 16 objects employed in the study. The object and grasps in panel **(A)** correspond to the second column of the Aperture subplot in panel **(C)**.

In Experiment 1, we employed 16 3D objects (4 shapes, 4 material configurations), each made of 10 cubes (2.53 cm^3^) of beech wood or brass. Objects with the same shape but different material configuration varied in mass (light wooden objects: 97 g, heavy wood/brass objects: 716 g) and mass distribution. These objects were the same, and were presented at the same orientations, as described in a previous study (Klein et al., 2020). For each of the objects, we selected pairs of grasps, one near-optimal and one sub-optimal, according to one of four grasp optimality criteria: natural grasp axis; optimal grasp aperture; minimum torque; optimal visibility. These criteria were mathematically defined as in our previous work (Klein et al., 2020). For each of these optimality criteria, we selected pairs of near-optimal and sub-optimal grasps on four of the 16 objects, while maintaining the other optimality criteria approximately constant across the grasp pair or counterbalanced across objects. Figure 2A shows one example object in which we selected one near-optimal and one sub-optimal grip with regard to grasp aperture. Figure 2B shows the optimality values for both grasps following each of the optimality criteria, and the difference in optimality between the two grasps. The difference in grasp optimality between pairs of grasps on all 16 objects for each of the four grasp optimality criteria is shown in Figure 2C. The selected grasp pairs were marked on the objects with colored stickers glued onto the objects’ surface. Thumb grasp locations were marked in either blue or green (randomly assigned to the near-optimal and sub-optimal grasps). Index finger locations were marked in yellow. All objects and selected grasp pairs are shown in Supplementary Figures 1–4.

#### Procedure

Experiment 1 consisted of a vision session followed by a grasping session. In each session, all objects were presented in random order. In a single trial of either session, participants were instructed to judge which of the two predefined grasps marked on the object was better. No specific definition of grasp quality was given to participants. In the vision session, no physical contact with the objects was allowed. Participants were instructed to imagine both grasp movements, one after the other in predefined but random order, and then verbally to report which of the two grasps they thought was best. In the grasping session, participants executed both grasps and verbally reported which grasp was best. Participants were instructed to perform imagined and real grasps with a precision grip, i.e., using only thumb and index finger.

Prior to the experiment, participants were introduced to the objects. All stimuli were laid out on a table, the meaning of the stickers was explained, and participants were instructed to view (but not touch) the objects from all angles. Participants were familiarized with the weight of beech wood and brass by placing a wooden bar and a brass bar in sequence on the participants’ outstretched palm for a few seconds. Between trials of both sessions, and between grasps within one trial, we ensured that participants did not see the experimenter manipulating the objects by asking participants to keep their eyes closed until the objects were positioned.

In the vision session, once the stimulus was positioned at the starting location at its specific orientation, participants (with their head positioned on the chinrest) were instructed to open their eyes and visually explore the object. The experimenter then instructed the participants to imagine executing one of the two grasps (the green or the blue, randomly selected by the experimental script). Participants were asked to imagine reaching toward the object, placing their thumb and index at the marker locations, picking up the object using a precision grip, and moving it to the target location. Once participants indicated that they had finished imagining the first grasp movement, the experimenter instructed them to imagine executing the other. Once they had finished imagining both grasps, they were asked to report which was best, with no time limit. Throughout the whole vision session, participants were instructed to keep both hands on their thighs to prevent them from attempting pantomime grasps.

In the grasping session, on each trial participants positioned their head on the chinrest, and their thumb and index finger at the starting location. Once the stimulus was positioned, participants opened their eyes and the experimenter specified which grasp to attempt first (green or blue, in random order to minimize trial order effects; Maiello et al., 2018). Once the participant reported they were ready, an auditory cue specified the beginning of the grasping movement. Participants were required to reach, grasp, pick up and move the object onto the goal location, and return their hand to the starting position, all within 3 s. Prior to the second grasp, the experimenter positioned the current object back on its starting location while participants kept their eyes closed. Once the object was positioned, the procedure was repeated for the second grasp.

### Experiments 2a and 2b

Experiment 2a was a replication of Experiment 1, except that we only employed a subset of the conditions and we recorded participants’ grasp movements during the grasping session using the ZED mini stereo camera. The primary purpose of Experiment 2a was thus to capture the video recordings necessary for Experiment 2b. Compared to Experiment 2a, Experiment 2b contained an additional experimental session where participants evaluated grasp quality from the videos of participants from Experiment 2a.

#### Stimuli

In Experiments 2a and 2b we employed only 6 objects out of the 16 employed in Experiment 1. This subset of conditions, shown in Supplementary Figure 5, was selected so that participants would be at chance performance in the vision condition and significantly above chance in the grasping condition.

#### Procedure

The procedure of Experiment2a was identical to that of Experiment 1, except with fewer conditions.

In contrast to Experiment 1 and 2a, Experiment 2b consisted of three sessions: first a vision, then a video session, followed by a grasping session. The first (vision) and third (grasping) sessions were identical to the first and second sessions of Experiment 2a. In the video session of Experiment 2b, participants were shown videos of participants from Experiment 2a grasping the objects at the predefined grasp locations. Participants across Experiments 2a and 2b were yoked: each participant from Experiment 2b saw and evaluated the grasps from only one participant from Experiment 2a. The videos were taken from the left lens of the Zed mini stereo camera. Participants sat in front of a computer monitor.

On each trial, a dialogue box informed subjects which of the two grasps (green or blue) they would be viewing first. Participants started the video with a mouse click. Once the first grasp video was shown, a dialogue box informed participants they would be viewing the second grasp, and once again, participants started the video. Each video was shown only once. After participants had viewed both videos, they reported, via mouse click, which of the two grasps was better.

### Analyses

Data analysis was performed in Matlab version R2018a. The dependent measure for all analyses was the proportion of trials in which the model-optimal grasp was rated as “better,” which we refer to as “Percent correct grasp optimality judgments.” Differences from chance performance and between group means were evaluated via unpaired and paired *t*-tests, as appropriate (*p* < 0.05 were considered statistically significant). We also report the 95% highest density interval (95% HDI) of the difference from chance or between group means, obtained via Bayesian estimation (Kruschke, 2013) using the Matlab Toolbox for Bayesian Estimation by Nils Winter. We compute effect size as μ−*Chance*/σ in case of differences from chance, and as μ_*G*1−*G*2_/σ_*G*1−*G*2_ in case of differences between group means. As we are interested in fairly moderate effects (Cohen, 1988), we define a region of practical equivalence (ROPE) on effect size from −0.4 to 0.4. In cases where no statistically significant difference is observed using frequentist hypothesis testing, we use this ROPE to assess how credible the null hypothesis is that there exist no meaningful differences from chance or between group means (Kruschke, 2011). In such cases, we report the effect size and percentage of its posterior distribution that falls within the ROPE.

## Results

### Experiment 1: Participants Can Report Whether Grasps Are Optimal Through Vision Alone, and Perform Better When Allowed to Execute the Grasps

**FIGURE 3:**
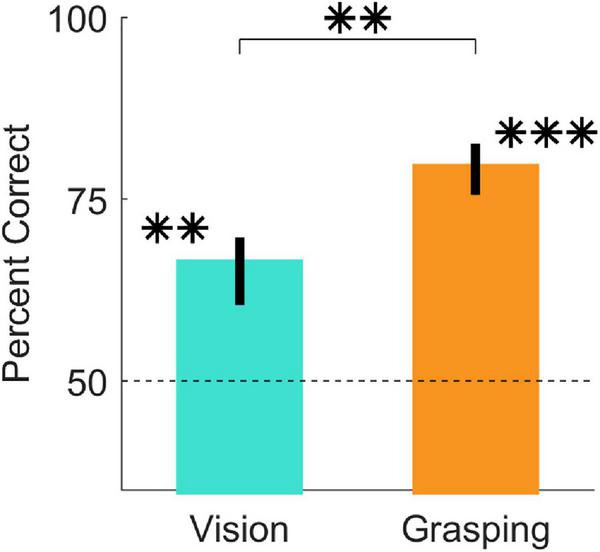
Judgments of grasp optimality using vision and grasping. Percent correct grasp optimality judgments for the vision session (left), and the grasping session (right), averaged across objects and participants. Error bars indicate 95% bootstrapped confidence intervals of the mean. Chance performance is 50% correct (dotted line). ^**^p < 0.01; ^***^p < 0.001.

In Experiment 1, we asked participants to perform imagined and real grasps on 16 objects and to report which of two predefined grasp locations was best. Figure 3, shows that participants were significantly above chance at judging grasp optimality when using vision alone [*t*(20) = 6.63, *p* = 1.9*10^–06^; 95% HDI = (11, 22)] and also when physically executing the grasps [*t*(20) = 15.79, *p* = 9.3*10^–13^; 95% HDI = (25, 33)]. Additionally, participant judgements significantly improved in the grasping session compared to the vision session [*t*(20) = 5.14, *p* = 5*10^–05^; 95% HDI = (8, 19)]. Percent correct grasp optimality judgments for individual objects, grouped by optimality conditions, are shown in Supplementary Figures 1–4. Note that we do not compare performance across optimality conditions as we did not equate difficulty across conditions, and even within the same condition task difficulty and performance could vary markedly.

### Experiment 2: Visual and Proprioceptive Information During Grasping Are Redundant for Evaluating Grasp Optimality

The results from Experiment 1 suggest that participants are better at judging grasp quality when they perform the grasp. However, Experiment 1 leaves open whether the performance increase is due to the sensorimotor or visual feedback during grasp. In Experiment 2, we tested whether visual cues from real grasp movements were sufficient to improve grasp optimality judgements. In Experiment 1, performance varied across optimality criteria and individual objects. Therefore, we selected the subset of conditions from Experiment 1 that showed the largest difference between the vision and grasping session.

**FIGURE 4:**
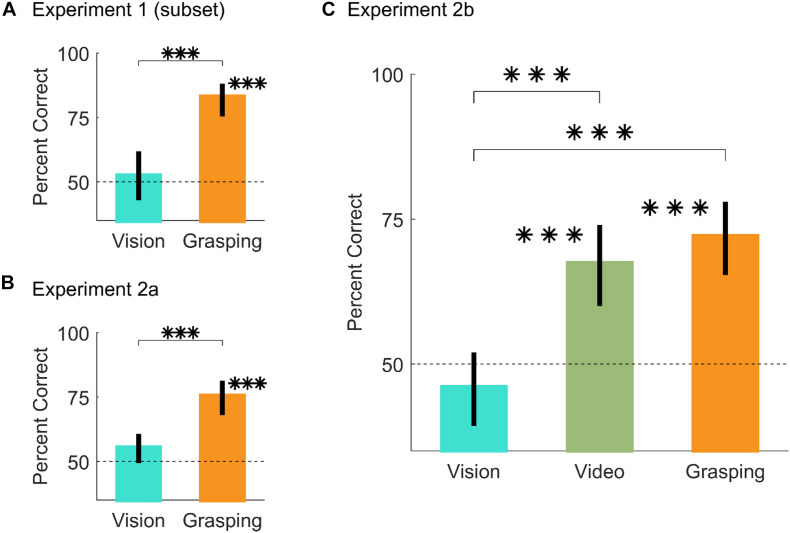
Results from Experiment 2. **(A,B)** Percent correct grasp optimality judgments for vision and grasping sessions, averaged across objects, and participants, for **(A)** the subset of conditions from Experiment 1 that drives the difference between vision and grasping, and **(B)** the same subset of conditions replicated in Experiment 2a. **(C)** Percent correct grasp optimality judgments for vision, video, and grasping sessions, averaged across objects and participants, for Experiment 2b. In all panels, error bars are 95% bootstrapped confidence intervals of the mean and chance performance is 50% correct (graydotted line). ^∗∗∗^*p* < 0.001.

Figure 4A shows that for these conditions, participants were at chance in the vision session [*t*(20) = 0.5, *p* = 0.62; 95% HDI = (-8, 13), effect size = 0.11, 88% in ROPE], above chance when physically executing the grasps [*t*(20) = 10.25, *p* = 2.1^∗^10^–09^; 95% HDI = (29, 40)], and performance in the grasping session was significantly improved compared to the vision session [*t*(20) = 4.81, *p* = 1.1^∗^10^–4^; 95% HDI = (19, 46)].

In Experiment 2a we replicated the results from Experiment 1 on this subset of conditions (Figure 4B): participants were at chance in the vision session [*t*(24) = 1.88, *p* = 0.073; 95% HDI = (-1, 12)], effect size = 0.38, 53% in ROPE), above chance when physically executing the grasps [*t*(24) = 7.27, *p* = 1.7^∗^10^–07^; 95% HDI = (18, 33)], and performance in the grasping session was significantly improved compared to the vision session [*t*(24) = 3.51, *p* = 0.0018; 95% HDI = (8, 32)]. During the grasping sessions of Experiment 2 we also recorded videos of the participants executing the grasps from approximately the participants’ viewpoint. Example videos are shown in the Supplementary Material.

In Experiment 2b, participants performed a vision, a video, and a grasping session on the same conditions employed in Experiment 2a. Critically, in the video condition participants judged grasp optimality on videos of participants from Experiment 2a grasping objects at optimal and sub-optimal locations.

Similarly to Experiment 2a, Figure 4C shows that in Experiment 2b, participants were at chance in the vision session [*t*(24) = −1.19, *p* = 0.25; 95% HDI = (-11, 4), effect size = −0.24, 81% in ROPE]. Conversely, participants were significantly above chance in both the video [*t*(24) = 4.58, *p* = 1.2^∗^10^–4^; 95% HDI = (10, 26)] and grasping sessions [*t*(24) = 6.41, *p* = 1.3^∗^10^–06^; 95% HDI = (15, 29)]. Compared to the vision session, performance was significantly improved in both the vision [*t*(24) = 4.23, *p* = 3^∗^10^–04^, 95% HDI = (10, 32)] and grasping sessions [*t*(24) = 6.35, *p* = 1.4^∗^10^–06^, 95% HDI = (17, 35)]. Finally, performance in the video and grasping sessions was equivalent [*t*(24) = 0.92, *p* = 0.36; 95% HDI = (-6, 16)], effect size = 0.18, 83% in ROPE). Percent correct grasp optimality judgments for individual objects and optimality conditions for both Experiments 2a and 2b are shown in Supplementary Figure 5.

It is also worth noting that in Experiment 2b, the grasping session was always performed last, so the exposure to grasp videos could conceivably have helped the judgments made in the grasping session. To test this possibility, we contrasted performance in the grasping session from Experiment 2b, with performance in the grasping session from Experiment 2a. Performance in the grasping sessions was equivalent across experiments [*t*(24) = 0.68, *p* = 0.50; 95% HDI = (-13, 7), effect size = 0.14, 90% in ROPE], suggesting that the grasp observation session did not improve the decision-making that comes out of physically performing the grasps.

## Discussion

When grasping objects guided by vision, humans select finger contact points that are near-optimal according to several physics- and biomechanics-based constraints (Kleinholdermann et al., 2013; Klein et al., 2020). Whether these constraints are explicitly computed in the brain is unknown. Here, we demonstrate that humans can explicitly judge which of two potential grasps on an object is best, based on each of these constraints.

In our study, participants could distinguish near-optimal from sub-optimal grasp locations using vision alone, i.e., without physically executing grasps, presumably using motion imagery. This well aligns with the notion that motor imagery, the mental simulation of a motor task, relies on similar neural substrates as action planning and execution. For example, it is well-established that simulated actions take the same time as executed ones (Decety et al., 1989; Jeannerod, 1995). This temporal similarity has also been shown in a task akin to the current study. Frak et al. (2001) asked participants to determine whether contact points marked on a cylindrical object placed at different orientations would lead to easy, difficult, or impossible grasps, without grasping the object. The time to make these estimates varied with object orientation and task difficulty, and closely matched the time taken to perform the grasps. These temporal matches hint that imagined and real actions might rely on similar neural computations. Indeed it has been shown that motor imagery recruits many of the same visuomotor areas of the brain, from early visual cortex (Pilgramm et al., 2016; Zabicki et al., 2016; Monaco et al., 2020), throughout the dorsal stream and the parietal lobe leading to primary motor cortex M1 (Hétu et al., 2013), that are directly involved in action planning and execution (Hardwick et al., 2018).

In Experiment 1 of our study, judgements of grasp optimality improved when participants were required to execute the grasps. What drove this improvement? Since the grasping session always came after the vision session, it is possible that the improvement in the grasping session could be due to participants learning the task or having gained familiarity with the objects. This is unlikely, however, since we did not provide participants with any feedback they might have used to learn the task, and we found no evidence of learning within the single sessions (see Supplementary Figures 6, 7). In the grasping sessions, participants were asked to grasp, lift and place the object at a goal location within 3s. However, they had unlimited time to plan the grasps prior to each trial. The planning stage in the grasping sessions was thus similar to the vision sessions. Therefore, in both sessions participants could build hypotheses about which grasp should be easier to execute, but only in the grasping sessions could they test these hypotheses against their own sensorimotor feedback. Specifically, if participants needed to make corrective changes once a movement had been initiated, it is possible that the difference between this event and the original motor intention could have reached consciousness and improved their judgements. However, previous research has shown that the recalibration of reach-to-grasp movements through haptic feedback occurs outside of perceptual awareness (Mon-Williams and Bingham, 2007). If participants could not consciously access the corrections to their original motor plans, crucial clues to indicate that a grasp was sub-optimal could be provided by tactile feedback from object slippage (Johansson and Westling, 1984), the need to apply greater grip forces than anticipated (Lukos et al., 2013), or proprioceptive feedback indicating awkward joint configurations (Rosenbaum et al., 2001).

Tactile and proprioceptive feedback were not the only sources of information that could have aided judgements in the grasping session. Participants could also visually assess the characteristics of their own movements, such as the speed and trajectory of the limb. These sources of visual information are known to play a strong role in grasp execution, as removing them changes the kinematics of grasping movements (Connolly and Goodale, 1999). Additionally, even if visual information from object roll during grasps does not influence the calibration of digit placement and force control (Lukos et al., 2013), lifting without visual feedback does impair fingertip force adaptation (Buckingham and Goodale, 2010; Buckingham et al., 2011). We therefore, wondered whether these sources of visual information alone could aid judgements of grasp optimality.

In Experiment 2, we indeed found that viewing videos of other participants grasping near-optimal and sub-optimal grasps was sufficient for observers to reach the same level of performance at reporting which grasp was best as when actually executing grasps. This does not mean that in the grasping sessions participants did not rely on tactile and proprioceptive feedback. It suggests instead, that visual and tactile/proprioceptive feedback may be redundant sources of information in evaluating grasp quality. This could help explain how humans are able to exploit action observation more generally. For example, humans are able to acquire useful information, such as object size and weight, by simply observing the movement kinematics of others (Bingham, 1987; Hamilton et al., 2007; Campanella et al., 2011; Ansuini et al., 2016; Podda et al., 2017). Additionally, observing others execute grasping tasks, particularly when they make errors, can improve one’s own grasping performance (Buckingham et al., 2014). Observing one’s own grasps, particularly when making errors, could thus link visual and tactile/proprioceptive information about grasp quality. This in turn would allow us to learn how best to grasp a novel object by simply looking at someone else grasping it.

### Limitations and Future Directions

Our findings reinforce the notion that motor imagery and action observation play an important role in learning complex motor tasks (Gatti et al., 2013). For this reason, motor imagery and action observation have also shown promise in aiding and strengthening motor rehabilitation techniques in a variety of neurological conditions (Sharma et al., 2006; Mulder, 2007; de Lange et al., 2008; Zimmermann-Schlatter et al., 2008; Malouin et al., 2013; Mateo et al., 2015). Within this context, our model-driven method of selecting optimal—and particularly sub-optimal—grasps could be used to guide and strengthen mental imagery and action observation techniques for motor rehabilitation. For example, patients could be made to imagine, observe, and execute grasps to object locations, selected through our modeling approach, which contain the most useful information for re-learning grasping movements.

In the vision session of Experiment 1, participants were above chance at judging grasp optimality for a majority of objects (10 out of 16), but not for all. This is likely due to our procedure for selecting pairs of near-optimal and sub-optimal grasps, which was not designed to equate task difficulty across objects and conditions. Yet what makes one pair of grasps more or less visually distinguishable in terms of optimality? This could be related to how humans encode the different constraints on grasp quality through vision. Misjudgments for the pair of grasps shown in Figure 2 might be due, for example, to inaccuracies in visually estimating the length of the grasp aperture with respect to the span of our hand, or to inaccuracies in judging the exact location of the object’s center of mass from visual 3D shape and material cues. A potential approach to test this hypothesis would be to extend our model to be image computable, i.e., able to derive the constraints on grasp selection directly from images of the objects. If the image processing stages of model were designed to mimic those of the human visual system (e.g., Chessa et al., 2016; Maiello et al., 2020) we might then expect the model to begin making the same misjudgments as human participants.

Even in the grasping sessions, however, in about 20% of trials participants did not agree with the model predictions. Does this mean participants could not access the information about grasp quality? We believe it is more likely that the model predictions are incomplete. For example, the model does not take into account that for some grasps with high torques, the objects might rotate and come to rest against a participants’ palm, stabilizing an otherwise potentially unstable grasp. Additionally, in the current work we did not account for the different importance given by individual participants to the different constraints (Klein et al., 2020). Inspect for example the data from the last panel of Supplementary Figure 2. Even though the selected sub-optimal grasp has much larger grasp aperture than the selected near-optimal grasp, the sub-optimal grasp has marginally less torque. Thus, if some participants gave much greater importance to the torque constraint, this might explain why their responses disagreed with model predictions. Finally, to avoid biasing participants toward our expected results, we explicitly abstained from providing participants with a precise definition of grasp quality. However, this means different participants might have interpreted the instructions differently. The concept of a “better” grasp may have been interpreted in many ways, such as easier, faster, more accessible, or more comfortable. It is thus possible that different criteria may lead to different judgments, and it will be important in future research to link these subjective dimensions of grasp quality to objective measures of grasping performance.

In Experiment 2, we found that action observation and action execution yielded equivalent accuracies. However, it remains unknown whether the accuracy is equivalent across these two conditions because an “action observation system” treats them equivalently, or because there are two systems operating, one based on action observation and one based on action execution and efference copy, for example, which can inform the decision-making process. Nevertheless, the videos from Experiment 2 could provide some further insight into which visual cues participants were exploiting to determine grasp optimality during action observation. For example, in Supplementary Video 1 an observer might notice the different time it takes the participant to lift the same object with two different grasps, or the slight wobbling of the object when grasped in the uncomfortable hand orientation. In Supplementary Video 2, a prominent visual cue comes from the initial failure in computing a successful trajectory to the sub-optimal grasp. A quantitative analysis of the grasping kinematics contained in these videos, using for example novel image based tracking algorithms (Mathis et al., 2018), may reveal the exact nature of the visual information human participants exploit during action observation and execution. The full video dataset from Experiment 2, as well as all other data from the study, are made freely available through the Zenodo repository (10.5281/zenodo.4382477).

Finally, our approach could be further developed to investigate the neural underpinning of visual grasp selection. The current study demonstrates how, through the computational framework described in Klein et al. (2020), we can identify grasps on arbitrary objects that isolate the individual components of grasp selection. In future studies, these unique grasp configurations could be employed as stimuli for targeted investigations of brain activity, making it possible to pinpoint the neural loci of each of the visuomotor computations underlying grasp planning and execution.

## Conclusion

We show that humans are capable of judging the relative optimality between different possible grasps on an object. For a majority of tested objects and grasp configurations, human participants could perform these judgments using vision alone, and refined their estimates of grasp quality using visual and proprioceptive feedback during grasp execution. These abilities are likely a key component of how humans visually select grasps on objects. Remaining challenges will be to identify where and how grasp optimality is learned and computed in the brain in order to guide grasp planning and execution.

## Data Availability Statement

The datasets and analysis scripts generated for this study can be found in the online Zenodo repository (10.5281/zenodo.4382477).

## Ethics Statement

The studies involving human participants were reviewed and approved by the Lokale Ethik−Kommission des Fachbereichs 06, LEK−FB06. The patients/participants provided their written informed consent to participate in this study.

## Author Contributions

GM, MS, LK, VP, and RF conceived and designed the study. GM and MS collected the data. GM analyzed the data. All authors wrote the manuscript.

## Conflict of Interest

The authors declare that the research was conducted in the absence of any commercial or financial relationships that could be construed as a potential conflict of interest.

## Supplementary Material

Supplementary Figure 1 Percent correct grasp optimality judgments, computed across participants from Experiment 1, for the four individual objects in the natural grasp axis conditions. In each panel, the top object demonstrates the approximate viewpoint of a participant. Thumb locations for selected grasps were marked on the objects in green or blue. The position of the opposing index finger was marked in yellow. The color code only served to mark and identify the grasps for participants, and was purposely unrelated to the grasp optimality. The middle and bottom object show the near-optimal and sub-optimal grasps, respectively, with the objects rotated solely for illustrative purposes, to better show the selected grasp locations. Error bars represent 95% bootstrapped confidence intervals.

Supplementary Figure 2 As Supplementary Figure 1, except for the four individual objects in the grasp aperture conditions.

Supplementary Figure 3 As Supplementary Figure 1, except for the four individual objects in the minimum torque conditions.

Supplementary Figure 4 As Supplementary Figure 1, except for the four individual objects in the object visibility conditions.

Supplementary Figure 5 As Supplementary Figures 1–4, except for the six individual objects employed in Experiments 2a and 2b.

Supplementary Figure 6 Participant performance (percent correct grasp optimality judgments) as a function of trial number for Experiment 1. Dots are the mean performance across observers; error bars represent 68% bootstrapped confidence intervals. Dotted lines are best fitting regression lines through the data. We found no significant correlations between trial number and task performance in either the Vision Session (*r* = 0.052, *p* = 0.34), nor the Grasping Session (*r* = −0.041, *p* = 0.45). There is thus no evidence that performance improved with more practice and more familiarity with the task and objects.

Supplementary Figure 7 Percent correct grasp optimality judgments as a function of trial number for the six objects from Experiment 2a. In the six small panels, dots are performance, computed across participants, for each of the six objects ordered as in Supplementary Figure 5. The final panel shows the average across objects. Error bars represent 68% bootstrapped confidence intervals. Dotted lines are best fitting regression lines through the data. The analysis in Supplementary Figure 6 averages across participants based on trial number. Thus, different objects contribute to the accuracy computed at each trial number. The large variance in accuracy across objects might thus hide learning effects. The smaller number of conditions in Experiment 2 allows us to investigate potential learning effects at the level of individual objects. We observed a significant positive correlation between trial number and task performance in the Vision Session only for the U shaped object (second panel, *r* = 0.93, *p* = 0.0069; all other correlations *p* > 0.1). Even for this object, this correlation was likely spurious, as it did not replicate in the data from Experiment 2b (*r* = 0.18, *p* = 0.74). Across objects (rightmost panel), we also found no significant correlations between trial number and task performance in either the Vision Session (*r* = 0.061, *p* = 0.72), nor the Grasping Session (*r* = −0.13, *p* = 0.44). There is therefore no evidence that performance improved with more practice and more familiarity with the task and objects.

Supplementary Video 1 Representative participant from Experiment 2a executing near-optimal (left) and sub-optimal (right) grasps for one object belonging to the natural grasp axis conditions.

Supplementary Video 2 Representative participant from Experiment 2a executing near-optimal (left) and sub-optimal (right) grasps for one object belonging to the object visibility conditions.

## References

[B1] Ansuini C. Cavallo A. Koul A. D’Ausilio A. Taverna L. Becchio C. (2016). Grasping others’ movements: rapid discrimination of object size from observed hand movements. *J. Exp. Psychol. Hum. Percept. Perform.* 42 918–929. 10.1037/xhp0000169 27078036 PMC5051634

[B2] Bingham G. P. (1987). Kinematic form and scaling: further investigations on the visual perception of lifted weight. *J. Exp. Psychol. Hum. Percept. Perform.* 13 155–177. 10.1037/0096-1523.13.2.155 2953848

[B3] Buckingham G. Goodale M. A. (2010). Lifting without seeing: the role of vision in perceiving and acting upon the size weight illusion. *PLoS One* 5:e9709. 10.1371/journal.pone.0009709 20300575 PMC2837753

[B4] Buckingham G. Ranger N. S. Goodale M. A. (2011). The role of vision in detecting and correcting fingertip force errors during object lifting. *J. Vis.* 11:4. 10.1167/11.1.4 21205872

[B5] Buckingham G. Wong J. D. Tang M. Gribble P. L. Goodale M. A. (2014). Observing object lifting errors modulates cortico-spinal excitability and improves object lifting performance. *Cortex* 50 115–124. 10.1016/j.cortex.2013.07.004 23953062

[B6] Campanella F. Sandini G. Morrone M. C. (2011). Visual information gleaned by observing grasping movement in allocentric and egocentric perspectives. *Proc. R. Soc. B Biol. Sci.* 278 2142–2149. 10.1098/rspb.2010.2270 21147800 PMC3107628

[B7] Cesari P. Newell K. M. (1999). The scaling of human grip configurations. *J. Exp. Psychol. Hum. Percept. Perform.* 25 927–935. 10.1037/0096-1523.25.4.927 10464939

[B8] Chessa M. Maiello G. Bex P. J. Solari F. (2016). A space-variant model for motion interpretation across the visual field. *J. Vis.* 16:12. 10.1167/16.2.12 27580091

[B9] Cohen J. (1988). *Statistical Power Analysis for the Behavioral Sciences*, 2nd Edn. Abingdon: Routledge.

[B10] Connolly J. D. Goodale M. A. (1999). The role of visual feedback of hand position in the control of manual prehension. *Exp. Brain Res.* 125 281–286. 10.1007/s002210050684 10229019

[B11] de Lange F. P. Roelofs K. Toni I. (2008). Motor imagery: a window into the mechanisms and alterations of the motor system. *Cortex* 44 494–506. 10.1016/j.cortex.2007.09.002 18387583

[B12] Decety J. Jeannerod M. Prablanc C. (1989). The timing of mentally represented actions. *Behav. Brain Res.* 34 35–42. 10.1016/S0166-4328(89)80088-9 2765170

[B13] Eastough D. Edwards M. G. (2006). Movement kinematics in prehension are affected by grasping objects of different mass. *Exp. Brain Res.* 176 193–198. 10.1007/s00221-006-0749-3 17072606

[B14] Frak V. Paulignan Y. Jeannerod M. (2001). Orientation of the opposition axis in mentally simulated grasping. *Exp. Brain Res.* 136 120–127. 10.1007/s002210000583 11204406

[B15] Gatti R. Tettamanti A. Gough P. M. Riboldi E. Marinoni L. Buccino G. (2013). Action observation versus motor imagery in learning a complex motor task: a short review of literature and a kinematics study. *Neurosci. Lett.* 540 37–42. 10.1016/j.neulet.2012.11.039 23206748

[B16] Goodale M. A. Meenan J. P. Bülthoff H. H. Nicolle D. A. Murphy K. J. Racicot C. I. (1994). Separate neural pathways for the visual analysis of object shape in perception and prehension. *Curr. Biol.* 4 604–610. 10.1016/S0960-9822(00)00132-9 7953534

[B17] Hamilton A. F. Joyce D. W. Flanagan J. R. Frith C. D. Wolpert D. M. (2007). Kinematic cues in perceptual weight judgement and their origins in box lifting. *Psychol. Res.* 71 13–21. 10.1007/s00426-005-0032-4 16311765 PMC2637436

[B18] Hardwick R. M. Caspers S. Eickhoff S. B. Swinnen S. P. (2018). Neural correlates of action: comparing meta-analyses of imagery, observation, and execution. *Neurosci. Biobehav. Rev.* 94 31–44. 10.1016/j.neubiorev.2018.08.003 30098990

[B19] Hétu S. Grégoire M. Saimpont A. Coll M.-P. Eugène F. Michon P.-E. (2013). The neural network of motor imagery: an ALE meta-analysis. *Neurosci. Biobehav. Rev.* 37 930–949. 10.1016/j.neubiorev.2013.03.017 23583615

[B20] Jeannerod M. (1995). Mental imagery in the motor context. *Neuropsychologia* 33 1419–1432. 10.1016/0028-3932(95)00073-C 8584178

[B21] Johansson R. S. Westling G. (1984). Roles of glabrous skin receptors and sensorimotor memory in automatic control of precision grip when lifting rougher or more slippery objects. *Exp. Brain Res.* 56 550–564. 10.1007/BF00237997 6499981

[B22] Klein L. K. Maiello G. Paulun V. C. Fleming R. W. (2020). Predicting precision grip grasp locations on three-dimensional objects. *plos Comput. Biol.* 16:e1008081. 10.1371/journal.pcbi.1008081 32750070 PMC7428291

[B23] Kleinholdermann U. Franz V. H. Gegenfurtner K. R. (2013). Human grasp point selection. *J. Vis.* 13:23. 10.1167/13.8.23 23887046

[B24] Kruschke J. K. (2011). Bayesian assessment of null values via parameter estimation and model comparison. *Perspect. Psychol. Sci.* 6 299–312. 10.1177/1745691611406925 26168520

[B25] Kruschke J. K. (2013). Bayesian estimation supersedes the t test. *J. Exp. Psychol. Gen.* 142 573–603. 10.1037/a0029146 22774788

[B26] Lederman S. J. Wing A. M. (2003). Perceptual judgement, grasp point selection and object symmetry. *Exp. Brain Res.* 152 156–165. 10.1007/s00221-003-1522-5 12879179

[B27] Lukos J. Ansuini C. Santello M. (2007). Choice of contact points during multidigit grasping: effect of predictability of object center of mass location. *J. Neurosci.* 27 3894–3903. 10.1523/JNEUROSCI.4693-06.2007 17409254 PMC6672423

[B28] Lukos J. R. Choi J. Y. Santello M. (2013). Grasping uncertainty: effects of sensorimotor memories on high-level planning of dexterous manipulation. *J. Neurophysiol.* 109 2937–2946. 10.1152/jn.00060.2013 23554435 PMC3680819

[B29] Maiello G. Chessa M. Bex P. J. Solari F. (2020). Near-optimal combination of disparity across a log-polar scaled visual field. *PLoS Comput. Biol.* 16:e1007699. 10.1371/journal.pcbi.1007699 32275711 PMC7176150

[B30] Maiello G. Paulun V. C. Klein L. K. Fleming R. W. (2018). The sequential-weight illusion. *I-Perception* 9:204166951879027. 10.1177/2041669518790275 30090321 PMC6077907

[B31] Maiello G. Paulun V. C. Klein L. K. Fleming R. W. (2019). Object visibility, not energy expenditure, accounts for spatial biases in human grasp selection. *I-Perception* 10:204166951982760. 10.1177/2041669519827608 30828416 PMC6390223

[B32] Malouin F. Jackson P. L. Richards C. L. (2013). Towards the integration of mental practice in rehabilitation programs. A critical review. *Front. Hum. Neurosci.* 7:576. 10.3389/fnhum.2013.00576 24065903 PMC3776942

[B33] Mateo S. Di Rienzo F. Bergeron V. Guillot A. Collet C. Rode G. (2015). Motor imagery reinforces brain compensation of reach-to-grasp movement after cervical spinal cord injury. *Front. Behav. Neurosci.* 9:234. 10.3389/fnbeh.2015.00234 26441568 PMC4566051

[B34] Mathis A. Mamidanna P. Cury K. M. Abe T. Murthy V. N. Mathis M. W. (2018). DeepLabCut: markerless pose estimation of user-defined body parts with deep learning. *Nat. Neurosci.* 21 1281–1289. 10.1038/s41593-018-0209-y 30127430

[B35] Monaco S. Malfatti G. Culham J. C. Cattaneo L. Turella L. (2020). Decoding motor imagery and action planning in the early visual cortex: overlapping but distinct neural mechanisms. *NeuroImage* 218:116981. 10.1016/j.neuroimage.2020.116981 32454207

[B36] Mon-Williams M. Bingham G. P. (2007). Calibrating reach distance to visual targets. *J. Exp. Psychol. Hum. Percept. Perform.* 33 645–656. 10.1037/0096-1523.33.3.645 17563227

[B37] Mulder T. H. (2007). Motor imagery and action observation: cognitive tools for rehabilitation. *J. Neural Transm.* 114 1265–1278. 10.1007/s00702-007-0763-z 17579805 PMC2797860

[B38] Nguyen V.-D. (1988). Constructing force- closure grasps. *Int. J. Robot. Res.* 7 3–16. 10.1177/027836498800700301

[B39] Paulun V. C. Gegenfurtner K. R. Goodale M. A. Fleming R. W. (2016). Effects of material properties and object orientation on precision grip kinematics. *Exp. Brain Res.* 234 2253–2265. 10.1007/s00221-016-4631-7 27016090 PMC4923101

[B40] Paulun V. C. Kleinholdermann U. Gegenfurtner K. R. Smeets J. B. J. Brenner E. (2014). Center or side: biases in selecting grasp points on small bars. *Exp. Brain Res.* 232 2061–2072. 10.1007/s00221-014-3895-z 24639066

[B41] Pilgramm S. de Haas B. Helm F. Zentgraf K. Stark R. Munzert J. (2016). Motor imagery of hand actions: decoding the content of motor imagery from brain activity in frontal and parietal motor areas: MVPA of imagined hand movements. *Hum. Brain Map.* 37 81–93. 10.1002/hbm.23015 26452176 PMC4737127

[B42] Podda J. Ansuini C. Vastano R. Cavallo A. Becchio C. (2017). The heaviness of invisible objects: predictive weight judgments from observed real and pantomimed grasps. *Cognition* 168 140–145. 10.1016/j.cognition.2017.06.023 28675815 PMC5585416

[B43] Roby-Brami A. Bennis N. Mokhtari M. Baraduc P. (2000). Hand orientation for grasping depends on the direction of the reaching movement. *Brain Res.* 869 121–129. 10.1016/S0006-8993(00)02378-7 10865066

[B44] Rosenbaum D. A. Meulenbroek R. J. Vaughan J. Jansen C. (2001). Posture-based motion planning: applications to grasping. *Psychol. Rev.* 108 709–734. 10.1037/0033-295X.108.4.709 11699114

[B45] Schot W. D. Brenner E. Smeets J. B. J. (2010). Posture of the arm when grasping spheres to place them elsewhere. *Exp. Brain Res.* 204 163–171. 10.1007/s00221-010-2261-z 20567809 PMC2892064

[B46] Sharma N. Pomeroy V. M. Baron J.-C. (2006). Motor imagery: a backdoor to the motor system after stroke? *Stroke* 37 1941–1952. 10.1161/01.STR.0000226902.43357.fc 16741183

[B47] Voudouris D. Brenner E. Schot W. D. Smeets J. B. J. (2010). Does planning a different trajectory influence the choice of grasping points? *Exp. Brain Res.* 206 15–24. 10.1007/s00221-010-2382-4 20820763 PMC2938418

[B48] Zabicki A. de Haas B. Zentgraf K. Stark R. Munzert J. Krüger B. (2016). Imagined and executed actions in the human motor system: testing neural similarity between execution and imagery of actions with a multivariate approach. *Cereb. Cortex* 27 4523–4536. 10.1093/cercor/bhw257 27600847

[B49] Zimmermann-Schlatter A. Schuster C. Puhan M. A. Siekierka E. Steurer J. (2008). Efficacy of motor imagery in post-stroke rehabilitation: a systematic review. *J. NeuroEng. Rehabil.* 5:8. 10.1186/1743-0003-5-8 18341687 PMC2279137

