# Corrigendum: Humans can visually judge grasp quality and refine their judgments through visual and haptic feedback

**DOI:** 10.3389/fnins.2022.1088926

**Published:** 2022-12-12

**Authors:** Guido Maiello, Marcel Schepko, Lina K. Klein, Vivian C. Paulun, Roland W. Fleming

**Affiliations:** ^1^Department of Experimental Psychology, Justus Liebig University Giessen, Giessen, Germany; ^2^Center for Mind, Brain and Behavior, Justus Liebig University Giessen, Giessen, Germany

**Keywords:** grasping, visual grasp selection, precision grip, shape, material, motor imagery, action observation

In the published article, there was an error in Figure 3. Figure 3B presents the correlation between change in grasping performance across conditions, and grasping performance in the vision condition. This is an instance of “circular analysis” (Makin and Orban de Xivry, 2019), in which we erroneously introduced a mathematical coupling between the variables in the regression analysis (Archie, 1981). For this reason, the correlation presented in Figure 3B is spurious and should not have been reported.

To rectify this error, a correction has been made to Figure 3 and its corresponding legend. Specifically, panel B of Figure 3 has been removed from the manuscript, and the following sentence has been removed from the caption of Figure 3: “(B) The grasping benefit (delta percent) as a function of the performance in the vision session, for each individual participant. The size of each dot represents the number of occurrences for each data point (one occurrence for small dots, two for large dots). Black line is best fitting linear regression line”.

The corrected Figure 3 and its caption appear below.

**Figure 3:**
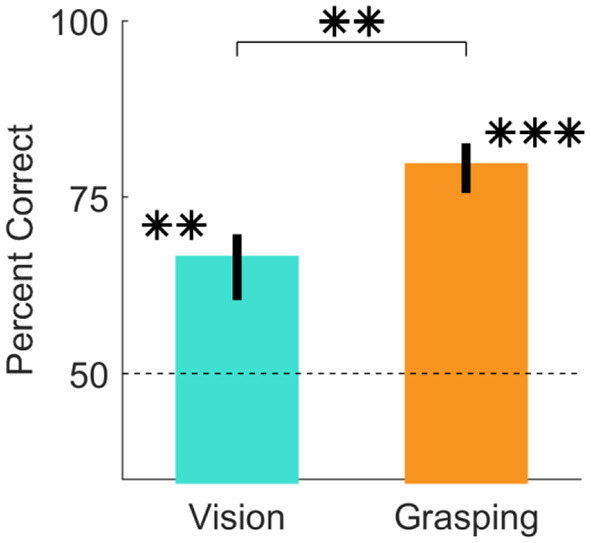
Judgments of grasp optimality using vision and grasping. Percent correct grasp optimality judgments for the vision session (left), and the grasping session (right), averaged across objects and participants. Error bars indicate 95% bootstrapped confidence intervals of the mean. Chance performance is 50% correct (dotted line). ^**^p < 0.01; ^***^p < 0.001.

To reflect this change, several minor corrections to citation of figures have been made as well as the deletion of footnote 1.

A correction has been made to Results, Experiment 1: Participants Can Report Whether Grasps Are Optimal Through Vision Alone, and Perform Better When Allowed to Execute the Grasps, paragraph 1, replacing the citation of ‘Figure 3A’ with ‘Figure 3’. Additionally, the sentence describing Figure 3B has been removed.

“In Experiment 1, we asked participants to perform imagined and real grasps on 16 objects and to report which of two predefined grasp locations was best. Figure 3 shows that participants were significantly above chance at judging grasp optimality when using vision alone [*t*(20) = 6.63, *p* = 1.9*10^−06^; 95% HDI = (11, 22)] and also when physically executing the grasps [*t*(20) = 15.79, *p* = 9.3*10^−13^; 95% HDI = (25, 33)]. Additionally, participant judgements significantly improved in the grasping session compared to the vision session [*t*(20) = 5.14, *p* = 5*10^−05^; 95% HDI = (8, 19)]. Percent correct grasp optimality judgments for individual objects, grouped by optimality conditions, are shown in Supplementary Figures 1–4. Note that we do not compare performance across optimality conditions as we did not equate difficulty across conditions, and even within the same condition task difficulty and performance could vary markedly.”

A correction has been made to Discussion, paragraph 3, in the first sentence the following has been removed: “and this improvement was strongest in participants who performed poorly using vision alone”.

“In Experiment 1 of our study, judgements of grasp optimality improved when participants were required to execute the grasps. What drove this improvement? Since the grasping session always came after the vision session, it is possible that the improvement in the grasping session could be due to participants learning the task or having gained familiarity with the objects. This is unlikely, however, since we did not provide participants with any feedback they might have used to learn the task, and we found no evidence of learning within the single sessions (see Supplementary Figures 6, 7). In the grasping sessions, participants were asked to grasp, lift and place the object at a goal location within 3s. However, they had unlimited time to plan the grasps prior to each trial. The planning stage in the grasping sessions was thus similar to the vision sessions. Therefore, in both sessions participants could build hypotheses about which grasp should be easier to execute, but only in the grasping sessions could they test these hypotheses against their own sensorimotor feedback. Specifically, if participants needed to make corrective changes once a movement had been initiated, it is possible that the difference between this event and the original motor intention could have reached consciousness and improved their judgements. However, previous research has shown that the recalibration of reach-to-grasp movements through haptic feedback occurs outside of perceptual awareness (Mon-Williams and Bingham, 2007). If participants could not consciously access the corrections to their original motor plans, crucial clues to indicate that a grasp was sub-optimal could be provided by tactile feedback from object slippage (Johansson and Westling, 1984), the need to apply greater grip forces than anticipated (Lukos et al., 2013), or proprioceptive feedback indicating awkward joint configurations (Rosenbaum et al., 2001)”.

The authors apologize for this error and state that this does not change the scientific conclusions of the article in any way. The original article has been updated.

## Publisher's note

All claims expressed in this article are solely those of the authors and do not necessarily represent those of their affiliated organizations, or those of the publisher, the editors and the reviewers. Any product that may be evaluated in this article, or claim that may be made by its manufacturer, is not guaranteed or endorsed by the publisher.
