# Supplementary material for: Humans Can Visually Judge Grasp Quality and Refine Their Judgments Through Visual and Haptic Feedback
Source: Front Neurosci. 2021 Jan 12;14:591898. doi: 10.3389/fnins.2020.591898 (PMC7835720; doi:10.3389/fnins.2020.591898)
Supplement: Supplementary Figure 1 — Percent correct grasp optimality judgments, computed across participants from Experiment 1, for the four individual objects in the natural grasp axis conditions. In each panel, the top object demonstrates the approximate viewpoint of a participant. Thumb locations for selected grasps were marked on the objects in green or blue. The position of the opposing index finger was marked in yellow. The color code only served to mark and identify the grasps for participants, and was purposely unrelated to the grasp optimality. The middle and bottom object show the near-optimal and sub-optimal grasps, respectively, with the objects rotated solely for illustrative purposes, to better show the selected grasp locations. Error bars represent 95% bootstrapped confidence intervals. [file Data_Sheet_1.PDF]

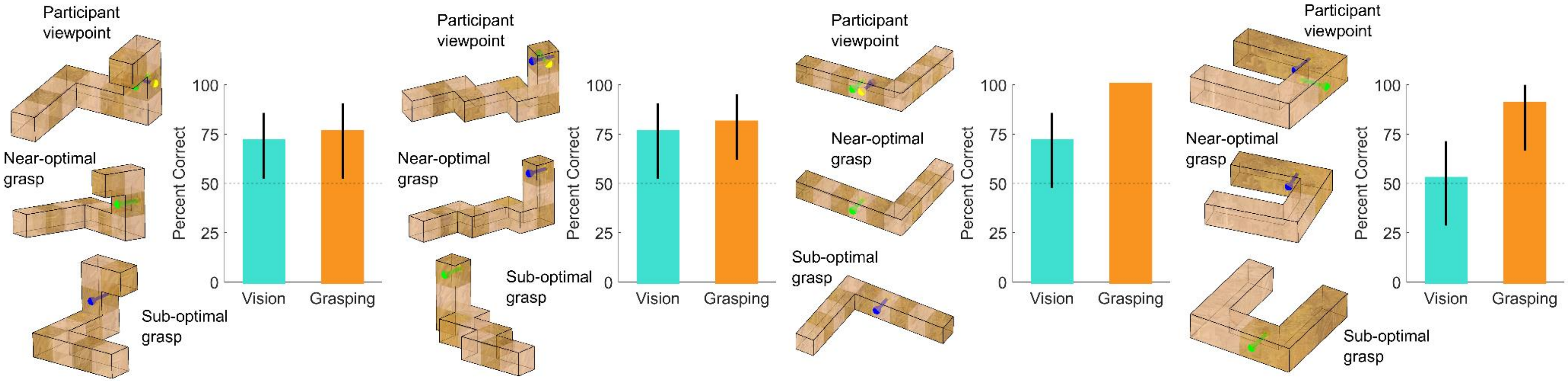

Supplementary Figure 1. Percent correct grasp optimality judgments, computed across participants from Experiment 1, for the 4 individual objects in the natural grasp axis conditions. In each panel, the top object demonstrates the approximate viewpoint of a participant. Thumb locations for selected grasps were marked on the objects in green or blue. The position of the opposing index finger was marked in yellow. The color code only served to mark and identify the grasps for participants, and was purposely unrelated to the grasp optimality. The middle and bottom object show the near-optimal and sub-optimal grasps respectively, with the objects rotated solely for illustrative purposes, to better show the selected grasp locations. Error bars represent 95% bootstrapped confidence intervals.

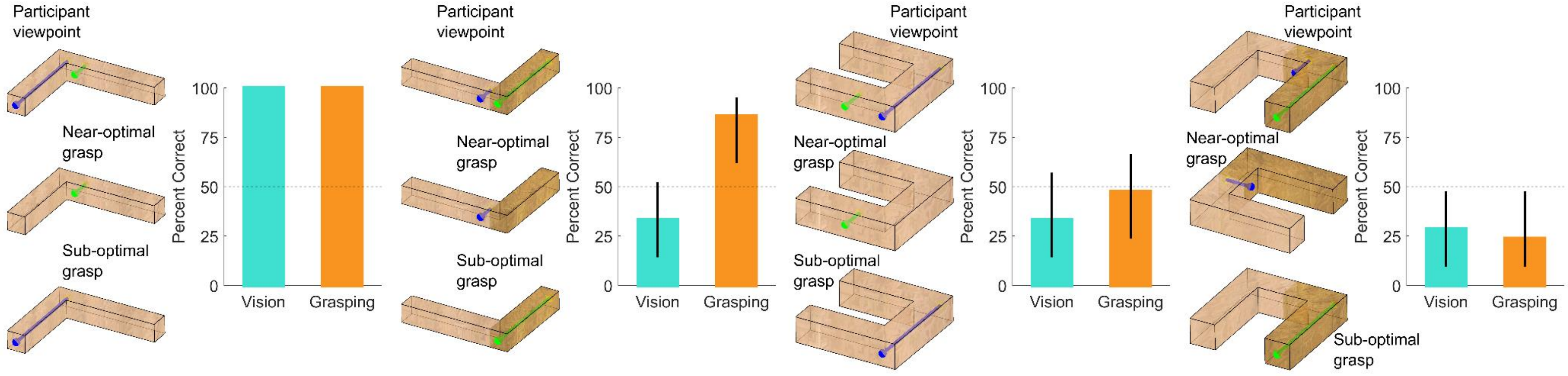

Supplementary Figure 2. As Supplementary Figure 1, except for the 4 individual objects in the grasp aperture conditions.

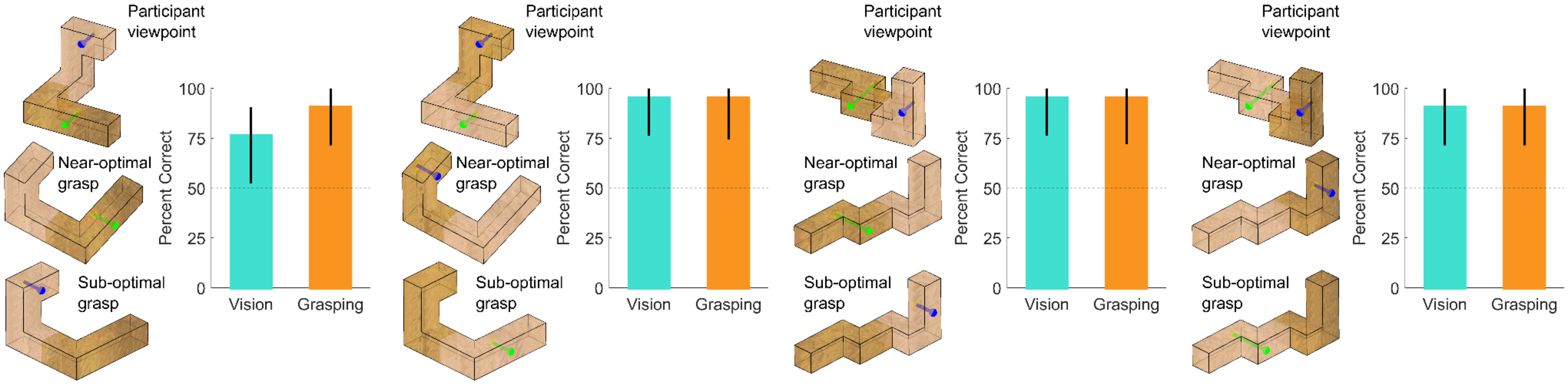

Supplementary Figure 3. As Supplementary Figure 1, except for the 4 individual objects in the minimum torque conditions.

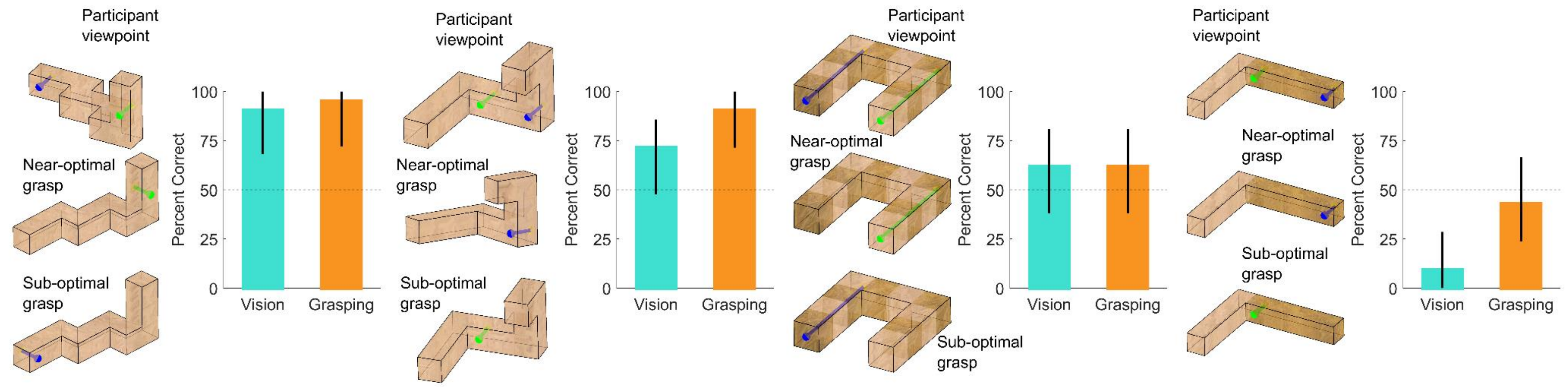

Supplementary Figure 4. As Supplementary Figure 1, except for the 4 individual objects in the object visibility conditions.

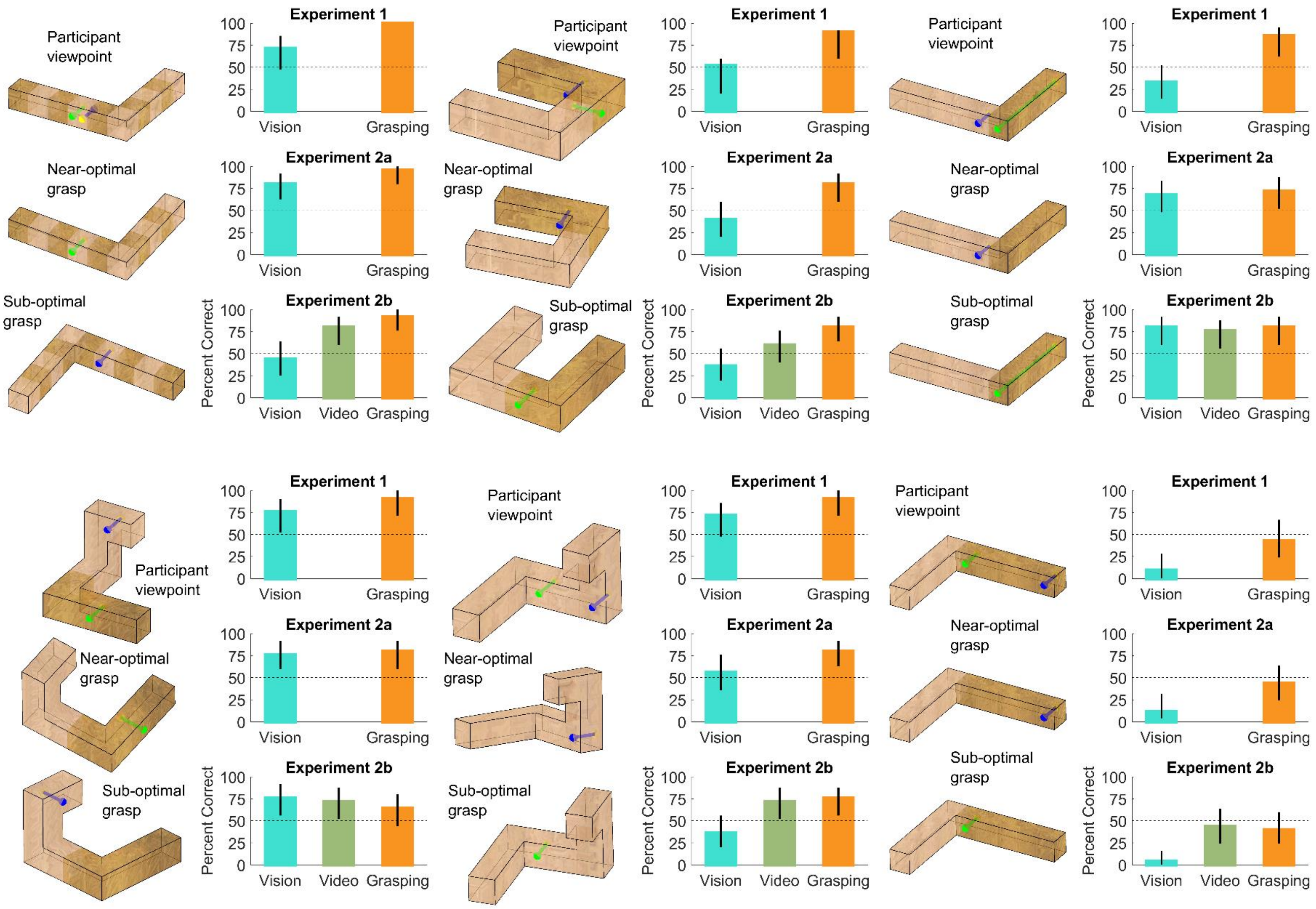

Supplementary Figure 5. As Supplementary Figures 1-4, except for the 6 individual objects employed in Experiments 2a and 2b.

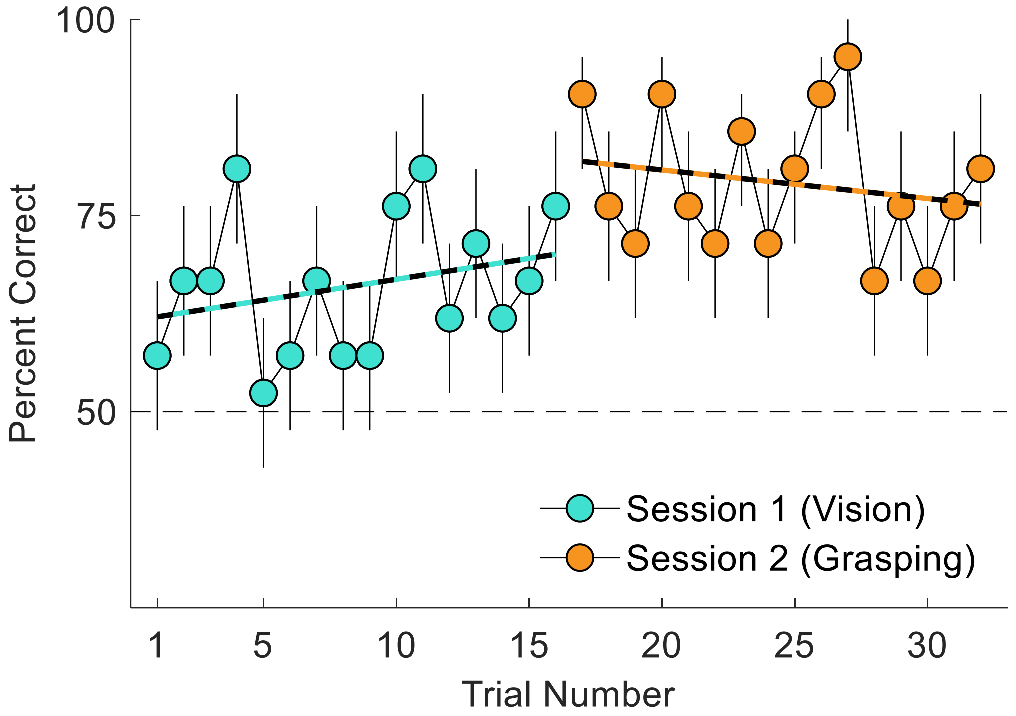

Supplementary Figure 6. Participant performance (percent correct grasp optimality judgments) as a function of trial number for Experiment 1. Dots are the mean performance across observers; error bars represent 68% bootstrapped confidence intervals. Dotted lines are best fitting regression lines through the data. We found no significant correlations between trial number and task performance in either the Vision Session ($r = 0.052$, $p = 0.34$), nor the Grasping Session ($r = -0.041$, $p = 0.45$). There is thus no evidence that performance improved with more practice and more familiarity with the task and objects.

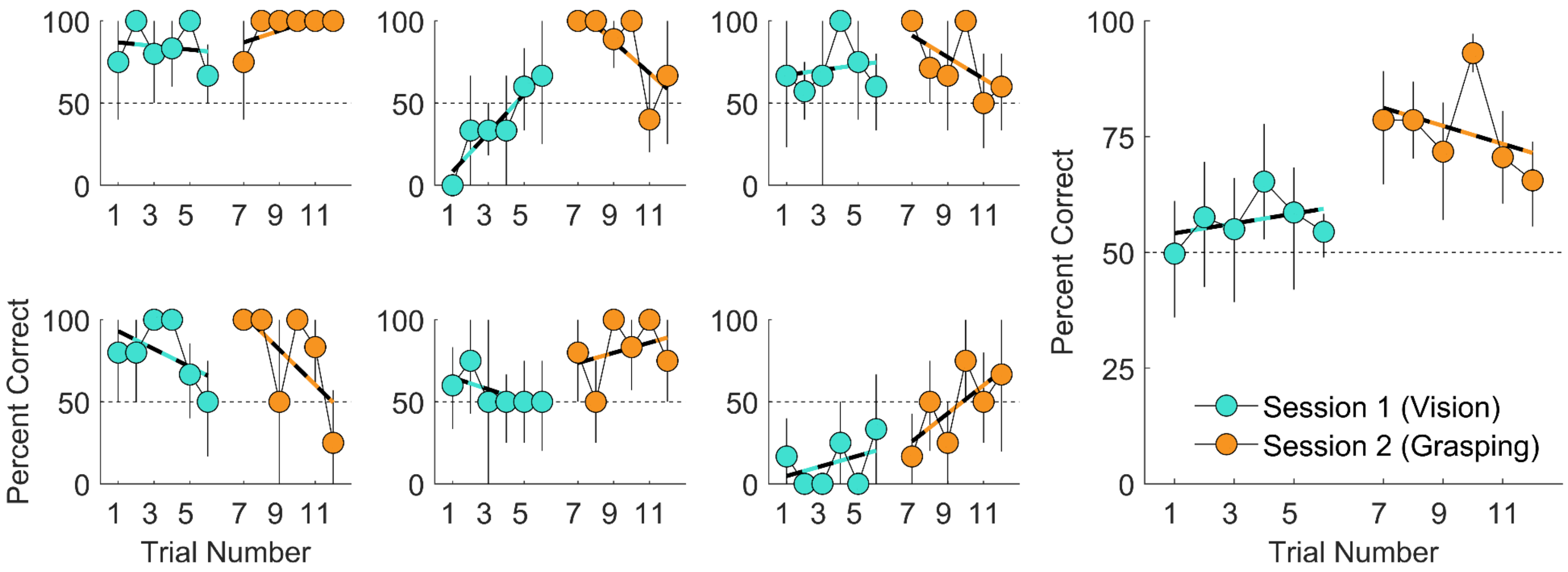

Supplementary Figure 7. Percent correct grasp optimality judgments as a function of trial number for the six objects from Experiment 2a. In the six small panels, dots are performance, computed across participants, for each of the six objects ordered as in Supplementary Figure 5. The final panel shows the average across objects. Error bars represent 68% bootstrapped confidence intervals. Dotted lines are best fitting regression lines through the data. The analysis in Supplementary Figure 6 averages across participants based on trial number. Thus, different objects contribute to the accuracy computed at each trial number. The large variance in accuracy across objects might thus hide learning effects. The smaller number of conditions in Experiment 2 allows us to investigate potential learning effects at the level of individual objects. We observed a significant positive correlation between trial number and task performance in the Vision Session only for the U shaped object (second panel, $r = 0.93$, $p = 0.0069$; all other correlations $p>0.1$). Even for this object, this correlation was likely spurious, as it did not replicate in the data from Experiment 2b ($r = 0.18$, $p = 0.74$). Across objects (rightmost panel), we also found no significant correlations between trial number and task performance in either the Vision Session ($r = 0.061$, $p = 0.72$), nor the Grasping Session ($r = -0.13$, $p = 0.44$). There is therefore no evidence that performance improved with more practice and more familiarity with the task and objects.
